# Maternal outcomes at mid-altitudes in anemic pregnant women: Evidence from the WOMAN-2 trial

**DOI:** 10.1371/journal.pone.0357033

**Published:** 2026-09-02

**Authors:** Projestine Muganyizi, Amy Brenner, Aasia Kayani, Eni Balogun, Folasade Adenike Bello, Gibson Kagaruki, Haleema Shakur-Still, Ian Roberts, Kiran Javaid, Mwansa Ketty Lubeya, Oladapo Olayemi, Rizwana Chaudhri, Raoul Mansukhani, Vwalika Bellington

**Affiliations:** 1 Department of Obstetrics and Gynaecology, University of Dar es Salaam, Mbeya College of Health and Allied Sciences, Dar es Salaam, Tanzania; 2 Clinical Trials Unit, London School of Hygiene & Tropical Medicine, London, United Kingdom; 3 Global Institute of Human Development, Shifa Tameer-e-Millat University, Islamabad, Pakistan; 4 Department of Obstetrics and Gynaecology, University of Ibadan College of Medicine Ibadan, Nigeria; 5 National Institute for Medical Research-Tanzania (NIMR); 6 Department of Obstetrics and Gynaecology, University of Zambia School of Medicine, Lusaka, Zambia; University of Global Health Equity, RWANDA

## Abstract

**Background:**

High residential altitude is associated with adverse pregnancy outcomes, yet most research has focused on elevations above 2500 meters. There is limited evidence on how altitude impacts maternal health below this threshold, where 99% of the global population resides. We investigated the effect of mid-altitudes on maternal outcomes among women with moderate to severe anemia who delivered in hospitals in four sub-Saharan and Asian countries.

**Methods:**

We analysed data from the World Maternal Antifibrinolytic-2 (WOMAN-2) trial, which recruited anaemic women admitted for vaginal delivery across 42 hospital sites in Nigeria, Pakistan, Tanzania, and Zambia. We compared women in mid-altitudes (1,000–2,499 m) with those at low-altitudes (< 1,000 m) using postpartum blood loss as the primary outcome. The key secondary outcome was clinical postpartum haemorrhage (PPH). Additional secondary outcomes included prenatal maternal health indicators (gestational age at labor onset, hypertensive disorders of pregnancy, baseline hemoglobin, severe anemia, and mean arterial pressure [MAP]), as well as intrapartum intervention indicators (assisted delivery, use of medical pain control, and use of oxytocin in labor). We used STATA version 18 for data analysis. We used classical logistic and modified Poisson regressions with cluster-robust standard errors to estimate the odds and risk ratios (RR) for dichotomous outcomes and linear regression with cluster-robust variance to estimate the β coefficients for the continuous outcomes.

**Results:**

Of 15,068 women, 4801 (31.9%) delivered at mid-altitude and 10,267 (68.1%) at low-altitudes. Delivery in mid-altitudes did not independently affect postpartum blood loss (β = 37.89 mL; 95% CI: −26.83–102.62), p = 0.243. Mid-altitude delivery compared to low-altitude was independently associated with a 22% lower risk of preterm delivery (aRR = 0.78; 95%CI: 0.73–0.83), p < 0.001, and a 26% lower risk of severe anaemia (aRR = 0.74; 95% CI: 0.68–0.82), p < 0.001. The use of medical pain control was 2.2 times higher (aRR = 2.20; 95% CI: 2.02–2.40), p < 0.001, and the Odds of assisted delivery were 46% more likely (aOR = 1.46; 95% CI: 1.20–1.78) at mid-altitudes compared to low-altitudes.

**Conclusion:**

Delivery at mid-altitudes was not independently associated with postpartum blood loss, but generally exhibited more favorable prenatal maternal health indicators and more intrapartum interventions than those delivering at low-altitudes.

## Introduction

High altitude has been associated with negative pregnancy outcomes [1–4]. A systematic review and meta-analysis by Grant et al. analyzed 59 studies involving 1,604,770 pregnancies at altitudes of 2500 meters or higher and found increased odds of low birth weight, small for gestational age, and preterm birth [1]. Another review focusing on maternal blood pressure at high altitudes (above 2500m) found that blood pressure, including gestational hypertension (but not preeclampsia), was higher compared to lower altitudes [3]. Many studies link these adverse outcomes to the body’s adaptation to chronic hypoxia and low barometric pressure at high elevations [4–8]. While fewer studies have explored the effects on maternal health specifically, some report increased maternal morbidity and mortality above 2500m, including hypertensive disorders of pregnancy, obstetric hemorrhage, and sepsis [3,7]. Other complications include polycythemia and related heart and lung issues, along with higher mortality [6,8–10].

Most research on the effect of altitude on pregnancy has been confined to altitudes at or above 2500 meters, despite the knowledge that 99% of the global population resides at altitudes below 2500 meters [5]. Thus, there remains a paucity of evidence from epidemiological studies to explain the effect of the variation in altitudes within the low to medium-altitude strata. Furthermore, the scant literature available remains heavily skewed toward the impact of altitude on fetal outcomes, with little attention paid to maternal outcomes. Studies on the effect of low to medium altitude on pregnancy outcomes in China, Austria, and Peru followed a similar trend as the studies in higher altitudes by focusing almost exclusively on fetal anthropometric features such as birth weights, small-for-gestation age, and prematurity [11,12]. In a population-based cross-sectional survey in Northwestern China among women with their infants born in 2010–2013, the focus was on birth weight, gestational age, and small for Gestational Age (SGA). According to this study, birth weight decreased by 6.4g, gestational age increased by 0.015 weeks, and small for gestational age (SGA) increased for every 100m increase in altitude, suggesting that low-to-medium altitude is possibly associated with some adverse fetal outcomes [11]. Another population-based study, which was conducted in the Austrian population residing at low-altitudes up to 1600 meters above sea level, focused on birth weight and concluded that it decreased by 150g per 1000 increase in altitude [12]. Nevertheless, none of these studies investigated how maternal outcomes were affected at medium altitudes.

This study explores the effect of mid-altitudes on postpartum blood loss, prenatal maternal health indicators, and intrapartum interventions.

## Materials and methods

### Study design and settings

We analysed data from the World Maternal Antifibrinolytic-2 (WOMAN-2), an international, randomized, double-blind, placebo-controlled trial that recruited 15,068 women with moderate-to-severe anaemia who were admitted for vaginal delivery in 42 hospitals and hospital units in Nigeria, Pakistan, Tanzania, and Zambia. Overall the trial started on 1^st^ November 2017 and was completed on 31^st^ October 2024. In Nigeria, the recruitment started 8^th^ March 2020 and ended on 19^th^ September 2023, in Pakistan it was from 24^th^ August 2019–19^th^ June 2023, in Tanzania it was from 2^nd^ March 2022–19^th^ September 2023 and in Zambia it started on 9^th^ January 2020 and ended on 16^th^ September 2023.

### Sample size calculation

To estimate the sample size for the primary study, a risk of PPH of 9% was assumed in the placebo group. We estimated that a trial with around 15,000 women would have 85% power to detect a 15% relative risk reduction in PPH (risk ratio 0.85) using a two-sided alpha of 5%. The methodological details about the WOMAN-2 study are published elsewhere [13,14]. The current analysis was based on the WOMAN-2 dataset.

### Sampling procedures

Participants in the WOMAN-2 trial were randomly assigned to receive either tranexamic acid or a matching placebo. Randomization codes for the investigational medicinal products were generated by an independent expert who was not involved in trial implementation. All women admitted for delivery at the 42 participating hospitals and hospital units were screened for eligibility, and hemoglobin concentrations were measured using the HemoCue Hb 201 + System.

Information about the trial was disseminated to pregnant women attending antenatal clinics through wall posters and information leaflets. Women with moderate anemia (Hb 70.0–99.0 g/L) or severe anemia (Hb < 70.0 g/L) on admission for delivery were considered eligible and enrolled after receiving detailed study information and providing written informed consent. Women younger than 18 years whose guardians did not provide consent were excluded from participation.

Eligible participants were randomized to receive either tranexamic acid or placebo (0.9% saline) as soon as possible after vaginal delivery and within 15 minutes of umbilical cord clamping. Standard hospital protocols for the prevention of postpartum hemorrhage (PPH) were maintained throughout the trial. Follow-up assessments included the diagnosis of PPH and estimation of total blood loss within 24 hours after delivery.

### Study variables

We collected altitude data for all the hospitals in the trial, specifically for this analysis. The average altitude for each delivery hospital's geographical area was obtained from a free online interactive map program at https://en-gb.topographic-map.com/ and verified by the local administrative authorities. In this analysis, women delivering at mid-altitudes (1,000–2,499 m above sea level) were compared with a reference group of women delivering at low altitudes (<1,000 m above sea level). The primary outcome was the volume of postpartum blood loss within 24 hours of delivery, measured in milliliters (mL).

The principal secondary outcome was a clinical diagnosis of PPH, defined as estimated blood loss of ≥500 mL or any amount of blood loss resulting in hemodynamic instability. Additional secondary outcomes included five indicators of maternal health before delivery: gestational age at the onset of labor (weeks), hypertensive disorders of pregnancy, baseline hemoglobin concentration (g/L), severe anemia (Hb < 70.0 g/L), and mean arterial pressure (MAP, mmHg). Three intrapartum intervention indicators were also assessed: assisted delivery, use of pain medication during labor, and administration of oxytocin during labor.

Mean arterial pressure was calculated using the formula:


$$\begin{array}{c} MAP\;=\;DBP\;+\;\text{1}/\text{3}(SBP\;-\;DBP), \end{array}$$

where SBP represents systolic blood pressure and DBP represents diastolic blood pressure.

### Training of the field team

Research personnel at all participating sites received standardized training through individual and group sessions that included PowerPoint presentations, role-playing exercises, instructional videos, and practical demonstrations of hemoglobin measurement procedures. Team members were also trained in the use of study instruments, including case report forms (CRFs), informed consent forms, and other trial-related documentation.

Training was guided by a standardized manual developed by the London School of Hygiene & Tropical Medicine (LSHTM) and adapted for implementation in each participating country. Before participating in the trial, all research staff completed Good Clinical Practice (GCP) training and obtained certification.

### Inclusivity in global research, refinement of data collection tools, and field manual

Research teams in each participating country reviewed all study materials and conducted stakeholder workshops involving potential participants and community members. These consultations aimed to ensure community voices are valued, promoting a sense of inclusion and importance in shaping the study. Feedback from these engagements was used to refine the study instruments and field manuals before implementation. Additional information regarding the ethical, cultural, and scientific considerations specific to inclusivity in global research is included in the supporting information (S3 File).

### Data collection, management, cleaning, and analysis

All trial data were managed in accordance with the WOMAN-2 Trial Data Management Plan (DMP) and stored within the trial master file. The DMP and its associated standard operating procedures complied with the policies and procedures of the London School of Hygiene & Tropical Medicine (LSHTM), the Clinical Trials Unit's operational requirements, and applicable regulatory standards.

The clinical database management system for the WOMAN-2 trial was developed in compliance with the International Council for Harmonization Good Clinical Practice (ICH-GCP) guidelines. The system utilized MySQL as the database management platform, while Hypertext Preprocessor (PHP) was used to develop the web-based user interface. The database was designed and maintained internally by the LSHTM Clinical Trials Unit.

Data were collected at each participating site and transmitted directly to the Clinical Trials Unit through the electronic database. In settings with limited internet connectivity, completed paper case report forms (CRFs) were submitted electronically via email. Routine data validation, quality checks, and cleaning procedures were conducted daily by the Clinical Trials Unit, with feedback provided to participating sites to ensure data completeness and accuracy.

We used STATA version 18 for data analysis. We conducted univariate analyses to examine the frequency and percentage distributions of the variables and to measure the central tendency and dispersion, including the mean, standard deviation, median, and interquartile range (IQR). Categorical data were compared between the mid-altitude and low-altitude using the Chi-square test. We used Student's t-test to compare means where the assumption of normal distribution was met, and Wilcoxon to compare continuous data if the assumption of normality was not met.

We applied classical logistic regression and modified Poisson regression with cluster-robust standard errors, accounting for clustering at the hospital level and including country as a fixed effect, as appropriate for modeling dichotomous outcomes. Classical logistic regression was used to estimate odds ratios (ORs) and 95% confidence intervals (CIs) when the outcome prevalence was 10% or less in the study population. Modified Poisson regression with cluster-robust standard errors was used to estimate risk ratios (RRs) and 95% confidence intervals (CIs) for dichotomous outcomes with a prevalence greater than 10% [15,16]. For continuous outcome variables, we performed linear regression with cluster-robust variance at the hospital level and country fixed effects, and results are presented as beta (β) coefficients with corresponding 95% CIs.

Both unadjusted and adjusted effect estimates are reported, including risk ratios (RRs), odds ratios (ORs), and beta (β) coefficients, together with their 95% CIs. Covariates incorporated into the multivariable regression models were selected on the basis of statistical significance observed in bivariate analyses (p ≤ 0.05), as well as their established clinical relevance, substantiated by prior literature and expert consensus.

In the multivariable analyses of dichotomous outcomes, adjustment covariates were selected according to the outcome under investigation. Hypertensive disease and preterm delivery (<37 weeks) were adjusted for maternal age. Severe anemia (hemoglobin <70 g/L) and oxytocin use during labor were adjusted for maternal age and previous cesarean section. Assisted vaginal delivery and use of pain control during labor were adjusted for maternal age, gestational age, previous cesarean section, and gravidity.

For continuous outcomes, blood loss was adjusted for oxytocin use during labor, mean arterial pressure (MAP), previous history of postpartum hemorrhage (PPH), maternal age, gestational age, previous cesarean section, gravidity, birth trauma, sepsis, placenta praevia, and antepartum hemorrhage. Baseline hemoglobin concentration was adjusted for maternal age and previous cesarean section, while MAP was adjusted for maternal age and gestational age.

### Ethical considerations

The WOMAN-2 protocol was approved by the National Health Research Ethics Committee of Nigeria (NHREC/01/01/2007–29/09/2019); the National Bioethics Committee of Pakistan (NBC-340); the National Institute of Medical Research of Tanzania (NIMR/HQ/R.8a/Vol.IX/3767); the University of Zambia Biomedical Research Ethics Committee (REF 001-04-19); and the London School of Hygiene and Tropical Medicine’s Ethics Committee (15194).

## Results

Of 15068, 4801 (31.9%) were delivered at the mid-altitudes and 10267 (68.1%) at the low-altitudes. The mean age of the participants, in years, was 27.2 ± 6.0 SD, and the mean gestational age in weeks was 37.4 ± 2.7. (Table 1).

**Table 1:** Distribution of participants’ delivery place and baseline characteristics by altitude level (n = 15068).

Variables	All	Low-altitude	Mid altitude	p-value
***Participants baseline characteristics***				
Age (yrs), mean (SD)	27.2(6.0)	27.1 (5.6)	27.4(5.4)	0.70
Gravidity, mean (SD)	3.1(2.2)	3.1, (2.1)	3.2 (2.3)	0.011
Gestational age (wks), mean (SD)	37.4(2.7)	37.3 (2.7)	37.7(2.7)	<0.001
Delivery location, n (%)	15068(100)	10267 (68.1)	4801 (31.9)	<0.001
Country of residency, n (%)				
Nigeria	1326(100)	1326 (100)	0	
Pakistan	11025 (100)	7623(69.1)	3402 (30.9)	<0.001
Tanzania	2029 (100)	1318(65.0)	711(35)	<0.001
Zambia	688 (100)	0	688(100)	

The median blood loss for all women was 280mL (IQR: 200- 350mL) and 730mL (IQR: 600- 925mL) for the women who developed PPH. The prevalence of hypertensive disorders in pregnancy was 7.6%, mean baseline Hb was 82.7 ± 11.8g/l, and mean MAP was 91.7 ± 10.7. Assisted delivery was conducted in 2.8% of women, pain control during labor was provided to 12.0% of women, and Oxytocin use was 51.1%. The diagnosis of clinical PPH in the mid-altitudes (6.7%) and the low-altitudes (6.9%) was comparable, with a p-value of 0.641. Overall, the median blood loss was 300 ml in mid-altitude compared to 280 ml in low altitude, p < 0.001. In the sub-analysis of blood loss among women who had lost at least 500 mL (PPH), the median blood loss was higher in mid-altitudes (800 mL) as compared to the low-altitudes (700 mL), p < 0.001. Compared to the low-altitudes, the mid-altitudes were also associated with statistically significantly fewer deliveries of preterm babies (at gestational age below 37 weeks), more assisted deliveries, and use of pain control medications in labor (Table 2).

**Table 2:** Comparison of key prenatal, intrapartum, and postnatal outcome indicators by altitude level (n = 15,068).

Variable	Overall	Low-altitude (N = 10267)	Mid-altitude (N = 4801)	p-value
Blood loss, all women (mL), median (IQR)	280 (200,350)	280(200,350)	300(200,355)	<0.001
Blood loss, PPH (mL), median (IQR)	730(600,925)	700(580,900)	800(600,980)	<0.001
Clinical PPH Diagnosis, n (%)	1035(6.9)	712(6.9)	323(6.7)	0.641
Hypertensive Disorders in pregnancy, n(%)	1146(7.6)	751(7.3)	395(8.2)	0.049
Baseline Hb (g/l), mean (SD)	82.7(11.8)	82.6(12.1)	83.1(11.4)	0.010
Severe anemia (<70g/l), n(%)	2079(13.8)	1518(14.8)	561(11.7)	<0.001
Mean arterial pressure(mmHg), mean (SD)	91.7(10.7)	92.1(10.8)	90.8(10.4)	<0.001
Delivery at gestation< 37 weeks, n (%)	3520 (23.4)	2583(25.2)	937(19.5)	<0.001
Assisted deliveries, n (%)	422(2.8)	254(2.5)	168(3.5)	<0.001
Medical pain control during labor, n(%)	1810(12.0)	892(8.7)	918(19.1)	<0.001
Oxytocin use in labor, n (%)	7693 (51.1)	5372(52.3)	2321(48.3)	<0.001

On multivariable analyses, the adjusted odds of hypertensive disease and the use of Oxytocin in labour were not statistically significant. The adjusted odds of assisted delivery were 46% higher in the mid-altitudes relative to the low-altitudes. The adjusted RR for medical pain control in labor in mid-altitudes was 2.2 times higher than in low-altitudes. The adjusted RR was 22% lower for preterm birth, and 26% lower for severe Anemia in the mid-altitude compared to the low-altitude (Table 3).

**Table 3:** The risk estimates of prenatal maternal health conditions and intrapartum interventions for the women who delivered at mid-altitudes, with the low altitude as the reference.

Outcome	Type of ratio	Unadjusted		Adjusted	
		RR/OR(95%CI)	p-value	RR/OR(95%CI)	p-value
Hypertensive disease	OR	1.14 (1.00-1.29)	0.049	1.11(0.98-1.26)	0.104
Severe anaemia (<70.0 g/L)	RR	0.79 (0.72-0.87)	<0.001	0.74(0.68-0.82)	<0.001
Oxytocin Use in Labour	RR	0.92 (0.89-0.96)	<0.001	1.00(0.97-1.04)	0.857
Assisted Delivery	OR	1.43 (1.17-1.74)	<0.001	1.46(1.20-1.78)	<0.001
Medical Pain Control	RR	2.20 (2.02-2.40)	<0.001	2.20(2.02-2.40)	<0.001
Preterm birth (<37weeks)	RR	0.78 (0.73-0.83)	<0.001	0.78(0.73-0.83)	<0.001

The adjusted baseline hemoglobin and blood loss for women who delivered at mid- and low altitudes were not statistically significant. There was a decrease of 1.36 mmHg in Mean Arterial Pressure (MAP) for women in the mid-altitudes compared to those in the low-altitudes (β) = −1.21(95%CI: −2.40 to −0.31), p < 0.012. (Table 4).

**Table 4:** Linear regression analyses of the association of delivery at mid-altitude with prenatal maternal health indicators and postnatal blood loss with the low-altitude as reference.

Outcome	Unadjusted (β),95%CI	p-value	Adjusted (β),95%CI	p-value
Blood loss, all women(mL)	10.32(3.85-16.79)	0.002	37.89(−26.83-102.62)	0.243
Blood loss, women with PPH (mL)	39.1(−4.10-82.3)	0.076	7.57(−34.19-49.32)	0.716
Baseline Hb (g/L)	0.53(0.12-0.94)	0.01	0.06(−0.19-0.31)	0.646
Mean arterial pressure (MAP/mmHg)	−1.30(−1.67 to −0.94)	<0.001	−1.36(−2.40 to −0.31)	0.012

## Discussion

This study aimed to evaluate the effect of mid-altitude on postpartum blood loss among anemic women delivering vaginally in hospital settings across four low and middle-income countries (LMICs) in sub-Saharan Africa and Asia—regions with a high burden of both maternal anemia and mortality [17–19]. In these settings, prophylactic uterotonic coverage—primarily oxytocin—exceeds 90% according to the WHO Multicountry Survey on Maternal and Newborn Health [18], and approximately half of the women in the WOMAN-2 trial received tranexamic acid, a proven intervention for reducing the life-threatening PPH [13,14,20].

Despite the use of prophylactic interventions, the crude analysis showed that women delivering at mid-altitudes experienced a median postpartum blood loss approximately 20 mL greater than those delivering at low altitudes. Although that difference is statistically significant, the adjusted analysis provided no evidence that delivery altitude was independently associated with the observed difference in the amount of postpartum blood loss. The observed slight difference appears to be largely explained by country-level effects, hospital-level variations, and residual confounding from factors such as oxytocin use during labor, mean arterial pressure (MAP), previous postpartum hemorrhage (PPH), maternal age, gestational age, prior cesarean delivery, gravidity, birth trauma, sepsis, placenta praevia, and antepartum hemorrhage, rather than by altitude itself.

We also examined differences in prenatal maternal health indicators between women delivering at mid- and low-altitude sites. These included MAP, the prevalence of hypertensive disorders of pregnancy, preterm deliveries (<37 weeks) leading to premature babies, baseline hemoglobin levels, and the occurrence of severe anemia. Overall, women at mid-altitudes presented with more favorable prenatal health indicators.

Specifically, MAP, a critical measure of vascular health and an early indicator of hypertensive disorders, was, on average, 1.11 mmHg lower among women at mid-altitudes. While the prevalence of hypertensive disorders was comparable across the study altitude levels, this lower MAP suggests a potentially reduced vascular risk profile among women at mid-altitude. Previous studies have reported varying outcomes in this regard. Bailey et al., analyzing data from over 600,000 women in Colorado, reported a higher incidence of hypertensive disorders at high altitudes (>2500 m), including gestational hypertension and preeclampsia [21]. Conversely, Grant et al. found that high altitude was associated with nearly a 50% reduction in preeclampsia risk [3]. Unlike our study, both analyses focused on higher-altitude populations and did not specifically examine anemic women. The relatively moderate MAP reduction in mid-altitudes in the current study may not be explained by hypoxic adaptive changes observed at altitudes above 2500m [22]. Thus, further research is needed to examine the association between altitude and cardiovascular health in pregnancy.

Another key finding was a 22% reduction in the risk of preterm birth among women giving birth at mid-altitudes. Preterm birth is multifactorial in origin, often linked to such factors as infections, maternal stress, poor nutrition, and hypertensive disorders. The literature on the relationship between altitude and preterm birth has been inconsistent. Grant et al., in a meta-analysis of 59 studies including over 1.6 million pregnancies, reported a higher risk of preterm birth at altitudes >2500 m [2]. In contrast, Levine et al. found no significant variation in preterm birth rates across low (0–1999 m), moderate (2000–2900 m), and high (3000–4340 m) altitude categories in an extensive cohort study in Peru [23]. Again, these discrepancies may be attributable to differences in altitude range and population characteristics.

All participants in the WOMAN-2 trial had moderate-to-severe anemia, confirmed at hospital admission. We compared the prevalence of severe anemia (Hb < 70.0 g/L) among women delivering at mid- and low-altitude sites and found that women delivering at mid-altitude had a 26% lower risk of severe anemia.

Although chronic hypoxia at high altitudes is known to stimulate physiological changes in hemoglobin concentration, the altitudes represented in this study may not have been sufficient to induce such pronounced adaptations. Furthermore, after adjustment for country, hospital, and other potential confounding factors, baseline mean hemoglobin concentrations were comparable between women delivering at mid- and low-altitude sites. These findings suggest that the degree of chronic hypoxia at mid-altitude may not have been sufficient to produce meaningful differences in baseline hemoglobin levels.

The prevalence and severity of anemia are influenced by a range of factors, including the burden of infectious diseases, agricultural practices that affect food availability and nutrition, access to healthcare services, and local social and cultural practices [8,11,12]. As this study did not comprehensively assess socioeconomic and nutritional determinants, we were unable to fully evaluate their contribution to the observed differences in anemia severity.

Finally, intrapartum interventions represented another area of interest. The use of assisted delivery and pain medications was higher in mid-altitudes than in low-altitudes, even after adjusting for country, hospital, and some residual confounding. The increased intervention rates at mid-altitudes could be partly explained by mild hypoxia-related physiological stress, possibly compounded by geographic, behavioural, and healthcare system factors that aren't fully captured even after adjusting for the confounding factors. Thus, this phenomenon is likely biopsychosocial, with biological, environmental, and systemic factors interacting.

A hypoxic environment can lead to physiological adaptations, such as increased hematocrit and blood viscosity, which may impair uteroplacental blood flow and oxygen delivery to the uterus [24]. Hypoxia also decreases Adenosine Triphosphate (ATP) levels in smooth muscles, activating ATP-sensitive K+ (KATP) channels and reducing intracellular Ca2 + levels. Thus, the diminution of ATP levels in chronic and acute hypoxia impairs oxytocin's signalling cascade, which relies on the availability of Ca2 + , ATP for second messenger systems, and actin-myosin cross-bridge, and the efficiency of ATP-sensitive K+ (KATP) channels. In addition, hypoxia can upregulate inducible nitric oxide synthase, increasing Nitric Oxide production, a potent smooth muscle relaxant, thereby antagonising oxytocin-induced contractions, among other possible mechanisms [25]. Consequently, labor may be less efficient, leading to a higher likelihood of interventions as a result of maternal exhaustion, fetal distress, and poor progress of labor.

### Strengths and limitations

To our knowledge, this is the first study to investigate the effect of mid-altitude residence on delivery outcomes among anemic women in low- and middle-income countries in Africa and Asia—regions that bear the highest global burden of maternal anemia and maternal mortality. The study draws on a well-maintained prospective cohort database with high-quality data collection, minimal missing data, and very limited loss to follow-up.

Our findings provide valuable insights into the potential influence of mid-altitude on antenatal health status, intrapartum care, and pregnancy outcome. Nevertheless, several limitations should be considered when interpreting the results. First, the study population consisted exclusively of anemic women who delivered in hospitals, and most participants received prophylactic interventions for postpartum hemorrhage (PPH). These factors may limit the generalizability of the findings to the broader population of pregnant women. However, given that nearly half of pregnant women in Sub-Saharan Africa and South Asia are affected by anemia and that institutional delivery rates continue to increase in these regions, the study remains highly relevant to its context [17–19].

A key limitation is the lack of data on participants’ socioeconomic and cultural backgrounds. Although we accounted for country-level differences, intra-country variations—such as rural versus urban settings, healthcare infrastructure, and access to medical services—can also affect delivery outcomes [10,11]. In mid-altitude regions, longer travel times and limited access to timely care may contribute to higher rates of labor interventions. Additionally, cultural attitudes toward pain and medical procedures vary by region, influencing the use of pain management and the likelihood of assisted deliveries. This is important, as both the altitude of residence and socioeconomic status can influence one another and may independently affect health outcomes [10,11].

Finally, individual participants’ residential altitudes were not available. Instead, we used the mean altitude above sea level for the geographic area where each delivery hospital was located as a proxy measure. Although area-level altitude may not perfectly reflect each participant's true residential altitude, we believe it provides a reasonable approximation because most women are likely to reside within the delivery hospital’s catchment area. Consequently, the observed associations are likely to reflect, at least in broad terms, the participants’ actual residential altitude. Moreover, recognizing that the study countries differed in landscape characteristics, we adjusted for country in the multivariable analyses to mitigate potential confounding. Future studies on the influence of residential altitude on maternal outcomes should seek to use the exact residential site altitude levels.

## Conclusions

In conclusion, mid-altitude delivery was not independently associated with postpartum blood loss. However, women delivering at mid-altitude exhibited more favorable pre-delivery health indicators than those delivering at low altitude, suggesting potential differences in maternal health profiles across altitude settings.

## Supporting information

S1 Table Hospitals with corresponding altitude values and the number of participants. (PDF)

S2 Table Maternal and labour characteristics in the low-and mid-altitude. Data presented as N(%) unless otherwise indicated.
(PDF)

S3 File Inclusivity in global research. (PDF)
