# Supplementary material for: Maternal outcomes at mid-altitudes in anemic pregnant women: Evidence from the WOMAN-2 trial
Source: PLoS One. 2026 Sep 2;21(9):e0357033. doi: 10.1371/journal.pone.0357033 (PMC13537555; doi:10.1371/journal.pone.0357033)
Supplement: Supplementary file 1 [file pone.0357033.s001.pdf]

S1Table. Hospitals with corresponding altitude values and the number of participants

**Delivery Hospital Site**	**Country**	**Meters Above Sea Level**	**Participants**
Holy Family Hospital Unit 1	Pakistan	499	264
Benazir Bhutto Shaheed Hospital	Pakistan	499	121
Ayub A Teaching Hospital	Pakistan	1096	408
Nishtar Hospital Unit 1	Pakistan	117	305
Sir Ganga Ram Hospital Units 1 - 4	Pakistan	215	595
Services Hospital Unit 1	Pakistan	215	121
Bolan Medical Centre Unit 3 and 4	Pakistan	1774	666
Bolan Medical Centre Unit 1 and 2	Pakistan	1774	576
Chandka SMBBMU Sheikh Zaid Woman Hospit-Unit 1	Pakistan	53	1,145
Chandka SMBBMU Sheikh Zaid Woman Hospit-Unit 2&3	Pakistan	53	673
Holy Family Hospital Unit 2	Pakistan	499	152
Federal Government Polyclinic	Pakistan	2015	88
MCH PIMS Unit 1 and 2	Pakistan	2015	681
Jinnah Hospital Lahore	Pakistan	215	727
Koohi Goth Hospital Karachi	Pakistan	95	276
Aziz Bhatti Shaheed Teaching Hospital -	Pakistan	229	132
Bahawalpur Victoria Hospital Units 1 &2	Pakistan	112	829
Civil Hospital Karachi Units 1 and 3 -	Pakistan	95	699
Jinnah Postgraduate Medical Centre Unit 1&2	Pakistan	95	856
Military Hospital	Pakistan	499	34
Adeoyo Maternity Hospital	Nigeria	190	242
Mother & Child Hospital Akure	Nigeria	333	250
Muhammad Abdullahi Wase Specialist Hosp	Nigeria	475	121
Women and Newborn Hospital	Zambia	1281	688

Ayub B Teaching Hospital	Nigeria	1096.00m	452
Ayub C Teaching Hospital	Nigeria	1096.00m	531
Services Hospital Unit 2	Pakistan	215.00m	172
Nishtar Hospital Unit 2	Pakistan	117.00m	310
Nishtar Hospital Unit 3	Pakistan	117.00m	212
State Hospital Oyo	Nigeria	299	230
Ilorin General Hospital	Nigeria	298	350
Muhimbili National Hospital	Tanzania	13	169
Mount Meru Regional Referral Hospital-	Tanzania	1400	278
Mwananyamala Regional Referral Hospital	Tanzania	22	730
Mbeya Zonal Referral Hospital	Tanzania	1712	40
Dodoma Regional Referral Hospital	Tanzania	1154	393
Ladoke Akintola University of Technolog	Nigeria	348	88
University of Medical Sciences Teaching	Nigeria	333	45
Temeke Hospital	Tanzania	29	155
Tumbi Hospital	Tanzania	138	85
Amana Hospital	Tanzania	51	179
