# Supplementary material for: Maternal outcomes at mid-altitudes in anemic pregnant women: Evidence from the WOMAN-2 trial
Source: PLoS One. 2026 Sep 2;21(9):e0357033. doi: 10.1371/journal.pone.0357033 (PMC13537555; doi:10.1371/journal.pone.0357033)
Supplement: Supplementary file 2 [file pone.0357033.s002.pdf]

S2.Table. Maternal and labour characteristics in the low-and mid-altitude. Data presented as N(%) unless otherwise indicated

Variables	Area of residence		
	Low-altitude	Mid-altitude	P-value
	N=10267	N=4801	
Episiotomy			
Age, mean years (SD)	27.1(5.6)	27.4(5.4)	<0.001
Gestational age (Weeks), Mean (SD)	37.3 (2.7)	37.7 (2.7)	<0.001
Delivery at Gestation age < 37 Weeks	3520(23.4)	2583 (25.2)	<0.001
Baseline Haemoglobin (g/L), Mean (SD)	82.7(11.8)	82.6(12.1)	0.010
Anaemia severity at baseline			
Moderate	8749(85.2)	4240(88.3)	<0.001
Severe	1518 (14.8)	561(11.7)	
Gravidity	3.1(2.1)	3.2(2.2)	
<5	8040(78.3)	3643(75.9)	0.001
5≤	2226(21.7)	1158(24.1)	
Previous Caesarean Section	445 (4.4)	259 (5.4)	0.010
Antepartum Haemorrhage	218 (2.7)	154 (3.2)	0.108
Blood loss in all women (mL), Median (IQR)	280 (200,350)	300(200,355)	<0.001
Blood loss in women with PPH (mL), Median(IQR)	700 (580,900)	800(600,980)	<0.001
Diagnosis of PPH	712 (6.9)	323(6.7)	0.641
Placental praevia	315 (3.1)	154 (3.2)	0.646
Hypertensive Disorders in Pregnancy	751(7.3)	395(8.2)	0.049
Mean Arterial Pressure, mmHg	92.1(10.8)	90.8(10.4)	<0.001
Assisted deliveries	254(2.5)	168 (3.5)	<0.001
Birth canal trauma	1378 (13.4)	812 (16.9)	<0.001
Medical Pain control during labour	892(8.7)	918 (19.1)	<0.001

Oxytocin use in labour	5372 (52.3)	2321 (48.3)	<0.001
Maternal deaths	11(0.11)	7 (0.15)	0.522
Maternal ICU admission	30 (0.29)	18 (0.38)	0.401
New infections	21(0.20)	6(0.13)	0.282
Cardiac dysfunction	20(0.19)	10 (0.21)	0.863
Hepatic dysfunction	2(0.02)	1(0.02)	0.956
Neurological dysfunction	3 (0.03)	3(0.06)	0.340
Sepsis	26(0.25)	4 (0.08)	0.029
