# Supplementary material for: Maternal outcomes at mid-altitudes in anemic pregnant women: Evidence from the WOMAN-2 trial
Source: PLoS One. 2026 Sep 2;21(9):e0357033. doi: 10.1371/journal.pone.0357033 (PMC13537555; doi:10.1371/journal.pone.0357033)
Supplement: Supplementary file 4 [file pone.0357033.s005.docx]

Date: 26^th^ June, 2026

Dear Editor,

Kindly find below the matrix indicating reviewers’ comments and our responses:

| **REVIEWER** | **COMMENT** | **RESPONSE** |
| --- | --- | --- |
| **Journal Editor** | Please ensure that your manuscript meets PLOS ONE's style requirements, including those for file naming. | Complied |
|  | Please include a complete copy of PLOS’ questionnaire on inclusivity in global research in your revised manuscript. Please upload a completed version of your questionnaire as Supporting Information when you resubmit your manuscript | The filled questionnaire has been uploaded as support information. The file is named S3Checklist. |
|  | Thank you for stating the following in the Acknowledgments Section of your manuscript:  “We acknowledge the valuable contributions to the WOMAN-2 Trial from the WOMAN-2 collaborators, as well as all colleagues and study participants in Pakistan, Nigeria, Tanzania, and Zambia. The WOMAN-2 trial was funded by The Gates Foundation (INV-007787) and The Wellcome Trust (WT208870/Z/17/Z).”  Please remove any funding-related text from the manuscript and let us know how you would like to update your Funding Statement. Currently, your Funding Statement reads as follows:  “The WOMAN-2 trial was funded by The Gates Foundation (INV-007787), https://www.gatesfoundation.org and The Wellcome Trust (WT208870/Z/17/Z), https://wellcome.org to London School of Hygiene and Tropical Medicine (LSHTM). The Funders did not play any role in study design, data collection, decision to publish or preparation of the manuscript.”  Please include your amended statements within your cover letter; we will change the online submission form on your behalf. | - Funding information has been deleted from the acknowledgement - The statement has been included in the cover letter, which is the same as in the Funding Statement section of the current online submission. |
|  | Thank you for uploading your study's underlying data set. Unfortunately, the repository you have noted in your Data Availability statement does not qualify as an acceptable data repository according to PLOS's standards. At this time, please upload the minimal data set necessary to replicate your study's findings to a stable, public repository (such as figshare or Dryad) and provide us with the relevant URLs, DOIs, or accession numbers that may be used to access these data. For a list of recommended repositories and additional information on PLOS standards for data deposition, please see https://journals.plos.org/plosone/s/recommended-repositories. | Minimal data has been uploaded to the FIGSHARE Repository: <https://doi.org/10.6084/m9.figshare.32794914> |
|  | Please include captions for your Supporting Information files at the end of your manuscript, and update any in-text citations to match accordingly. Please see our Supporting Information guidelines for more information: http://journals.plos.org/plosone/s/supporting-information. | Captions included:   - S1Table.Hospitals with corresponding altitude values and the number of participants - S2Table. Maternal and labour characteristics in the low-and mid-land. Presented as N(%) unless otherwise indicated - S3Checklist. Inclusivity in Global Health Research |
|  | If the reviewer comments include a recommendation to cite specific previously published works, please review and evaluate these publications to determine whether they are relevant and should be cited. There is no requirement to cite these works unless the editor has indicated otherwise. | No new citation recommendation was received. Supporting information is available at: <https://doi.org/10.6084/m9.figshare.32794914> |
|  | Please review your reference list to ensure that it is complete and correct. If you have cited papers that have been retracted, please include the rationale for doing so in the manuscript text, or remove these references and replace them with relevant current references. Any changes to the reference list should be mentioned in the rebuttal letter that accompanies your revised manuscript. If you need to cite a retracted article, indicate the article’s retracted status in the References list and also include a citation and full reference for the retraction notice. | Checked for Retraction. No retracted reference was found. |
| **Additional Editor’s comments** | Abstract 1) The conclusion appears somewhat overstated. The improvements in hemoglobin, reduced preterm birth, and lower severe anemia at mid-altitudes are supported by the results; however, MAP was lower, which complicates the interpretation of better prenatal health overall.  In addition, although blood loss was statistically higher, there was no corresponding difference in the prevalence of PPH. Therefore, it is difficult to conclude that mid-altitude exposure is associated with a clinically meaningful increase in blood loss, and this distinction should be reflected more cautiously in the conclusion. | We reviewed the analysis by accounting for clustering at the hospital level and including country as a fixed effect, as appropriate for modeling dichotomous and continuous outcomes. From the reanalysis:   - The significance of lower MAP persisted, which means a lower risk of cardiovascular disease, thus supporting overall better prenatal health and a lower risk of cardiovascular disease, such as pre-eclampsia among women in mid-altitude areas. - After the reanalysis, mid-altitude was not independently associated with increased blood loss, hence the conclusion has been revised accordingly. |
|  | Methods 2) The manuscript does not clearly follow standard methodological subheadings (e.g., design, data collection, sampling and sample size, and data analysis).  In particular, the adequacy of the existing sample powered to answer the stated objectives and how the sampling framework aligns with the altitude comparison is not sufficiently discussed. | - The methodology section and the manuscript as a whole has been reformatted to comply with PLOS requirements. |
|  | 3) It is not clearly stated whether women’s altitude exposure was measured at the individual level or assigned as a proxy based on hospital location. If altitude was derived from hospital geographical location, please clarify the rationale and methodological approach used to link hospital altitude to individual maternal exposure during pregnancy, and address the potential for exposure misclassification | - Women’s altitude exposure was based on the mean altitude at the delivery hospital's geographic location, as explained in the Materials and Methods **(L125-L131).** - Further, it has been acknowledged that delivery altitude could differ from the residential altitude, since most such deliveries come from the hospital catchment area; we reasonably took it as a proxy for individual maternal residential exposure **(L365-L371).** - The entire document has been revised to read as *delivery altitude* rather than residential altitude. |
|  | 4) The analysis of blood loss outcomes does not appear to account for baseline anemia status in the adjustment models, which should be clarified or justified. Results | - Yes, we did not control for anemia because Anemia was a criterion for enrolment of all the women in the study. All women in this study were anemic. |
|  | 5) In Table 1, the chi-square values are not presented | - We have revised Table 1 to include the chi-square/t-test p-values where appropriate as advised (Table 1). |
|  | 6) The content in Lines 176–181 and 194–198 appears to describe methodological procedures and would be more appropriately placed in the Methods section rather than the Results section. | - This has been revised such that all the misplaced methodological information has been relocated to the Materials and Methods section **(L185-L310)** |
| Reviewer #1 | 1). The manuscript states that the women were categorized as having a mid- or low attitude based on the altitude of the hospital where they gave birth. The part of the method section from line number 119-121, including the sentence “The average altitude for each delivery hospital's geographical area was obtained from a free online interactive map program,” confirms this. And the results were repeatedly interpreted as effects of “living” or “residing” at mid-altitude. As someone who grew up and lived in Africa, I knew that women's residential areas and delivery hospital addresses could vary widely because of a lot of reasons: some culture encourage women to give birth in area were they could get better support such as parents house, in-laws house, older sibblings house etc. it is also common for pregnant women with suspected complication outcomes to be referred to hospitals far away from their residential area. Thus, it will be problematic to conclude that the hospital area altitude reflects the women’s residential area. It is better to clarify this and make appropriate modifications | - We have revised the entire document to ensure the altitudes are referred to as places of delivery/birth rather than the places of residence. - In the discussion section, the altitudes for hospital location are discussed as a proxy to residencial altitude since most participants who gave birth in a hospital are expected to reside within the hospital catchment area. However, this assumption is acknowledged in the study limitations paragraph and suggested for future studies to use the individual participant’s residential location altitude for better outcomes **(L365-L372).** |
|  | 2) The other concern is the hierarchical structure and clustering consideration in the analysis. It seems the authors used a standard regression model without multilevel adjustment or clustering. Women are nested within hospitals and countries, yet the analysis appears to use standard regression models without multilevel adjustment or cluster-robust inference. Since altitude is strongly confounded by country/site, and clinical practice and intrapartum interventions differ between hospitals, failure to account for clustering may substantially underestimate standard errors and inflate statistical significance. The manuscript could be benefited from clarify about clustering by hospital/site, using mixed effects or other related models, use hospital/site as a random effect, etc., something that considers different hospitals, sites, and countries | - The analysis has been revised, taking into consideration the hierarchical structure and clustering [cluster 1: Individual participant; Cluster 2: Country; Cluster 3: Hospital site - Based on the hierarchy, we applied classical logistic regression and modified Poisson regression with cluster-robust standard errors, accounting for clustering at the hospital level and including country as a fixed effect to model dichotomous outcomes **(L185-L199).** - The analysis is justified since we have a good number of hospital sites (#42), and our interest was to apply the results to the population rather than studying variations among hospitals. - Likewise, for continuous outcome variables, we performed linear regression with cluster-robust variance at the hospital level and country fixed effects. - The analysis is explained in the Materials and Methods section (L185-L199), and the revised results are in Tables 3 & 4. |
|  | 3)The third major concern is altitude here is likely a proxy for country, residency, hospital type, referral pathways, ethnicity, nutrition, healthcare access, and culture. Although the lack of socioeconomic data is acknowledged in the manuscript, its implications are underestimated. The variable pain control, assisted delivery, oxytocin use, etc., could be affected by the above factors. For example, the 2.5-fold increase in pain medication certainly reflects variation in institutional practice rather than physiologic altitude effects. It would be better to interpret the findings carefully after distinguishing biologically plausible findings from healthcare-system-associated findings. Some explanation of the confounding by facility level clinical practice and adusting for hospital characteristics(if possible) also could benefit the manuscript.  The interpretation of clinical significance of blood loss ( including the phrase “this difference is both statistically and clinically meaningful”), and lower preterm birth risk requires careful revision with evidence-based justifications.    Terminology issue: the incidence rate ratio is used for a cross-sectional binary outcome with a modified Poisson regression analysis. Consider replacing it with the risk ratio(RR). Edite the word “Linear Logistic regression” in table 4. The phrase: “assumption of normality was not met based on the Z-test” is not clear. | - The analysis has been revised as explained above to take care of the confounding effect by country and hospital. - In the reanalysis, some residual confounders have been controlled. However, socioeconomic variables were not available; hence, the acknowledgement is made in the limitation section **(L356-L399).** - After the reanalysis above, blood loss was not independently associated with altitude level; hence, its discussion has been revised. - The low preterm birth rate in mid-altitudes in the current study could be linked to altitude. The association Lof altitude with preterm birth has resulted in inconsistent findings in previous studies as explained in the discussion section **(L297-L305)**. - These terminologies have been revised. The incidence rate ratio have been correctly replaced by Risk ratios (RR). “Linear Logistic regression” has been corrected to read “linear regression” (**L185-199).** - The phrase “assumption of normality was not met based on the Z-test” is corrected to read as “assumption of normality was not met.” (L178-L184) |
|  | 4). Confusing : “Baseline hemoglobin was 0.63 g/L higher” somewhere else hemoglobin values are reported around 82 g/L. Give it a look.  Minor Comments The manuscript would benefit from language editing and consistency in phrases like “mid altitude,” “mid-altitude,”“medium altitude.” Asia countries should be Asian countries | - The language has been corrected throughout the document. Mid-altitude is termed mid-altitude and low altitude as low-altitude throughout the document. |
