# Supplementary material for: Maternal outcomes at mid-altitudes in anemic pregnant women: Evidence from the WOMAN-2 trial
Source: PLoS One. 2026 Sep 2;21(9):e0357033. doi: 10.1371/journal.pone.0357033 (PMC13537555; doi:10.1371/journal.pone.0357033)
Supplement: Supplementary file 5 [file pone.0357033.s006.docx]

Date: 25^th^ July, 2026

Dear Editor,

Kindly find below the matrix indicating reviewers’ comments and our responses:

| **REVIEWER** | **COMMENT** | **RESPONSE** |
| --- | --- | --- |
| Editor | 1. Please upload a Response to Reviewers letter which should include a point by point response to each of the points made by the Editor and / or Reviewers. (This should be uploaded as a 'Response to Reviewers' file type.) Please follow this link for more information: <http://blogs.PLOS.org/everyone/2011/05/10/how-to-submit-your-revised-manuscript/>  2. It is important that you include a cover letter with your manuscript.  Please ensure that this letter is addressed specifically to PLoS ONE.  A cover letter has been included that addresses the following:  * why this manuscript is suitable for publication in PLoS ONE. * how does your paper provide a worthwhile addition to the scientific literature? * how does your paper relate to previously published work?  * which types of scientists do you believe will be most interested in your study? | The Response to Reviewers letter has been uploaded, which includes our responses to points by the Editor and Reviewer #1.  A cover letter has been uploaded with details of the outlined points. |
|  |  |  |
| Reviewer #1 | 1. Covariate selection description It says “Covariates included in the multivariable regression models were selected based on statistical significance in the bivariate analyses (p ≤ 0.05) and their established clinical relevance...” Could you revise this wording? It's not the best way to describe it. | Thank you for the comment. The statement has been refined to read as, *“Covariates incorporated into the multivariable regression models were selected on the basis of statistical significance observed in bivariate analyses (p ≤ 0.05), as well as their established clinical relevance, substantiated by prior literature and expert consensus.”***[L196-199]** |
|  | 2. Residual confounding Altitude remains strongly correlated with country. Some countries are either mainly mid-altitude or low-altitude. Could you discuss this limitation a little more explicitly? | We agree with the observation. We have added a statement that reads as, “Moreover, recognizing that the study countries differed in landscape characteristics, we adjusted for country in the multivariable analyses to mitigate potential confounding” to the limitations in the Discussion section **[L374-377]** |
|  | 3. Clarification of the sample size From my understanding, no additional sample size calculation was performed because this was a secondary analysis of an existing dataset. Could you clarify that, please? | We have added a short sentence to the Sample Size Calculation subsection which states: *“The current analysis was based on the WOMAN-2 dataset”* **[L106].** |
|  | 4. Typo errors including missing spaces, letters, and some inconsistent capitalizations These issues are minor and can be addressed without additional analyses. | The document was cross-checked for typos and corrected. |
