# Supplementary material for: Maternal outcomes at mid-altitudes in anemic pregnant women: Evidence from the WOMAN-2 trial
Source: PLoS One. 2026 Sep 2;21(9):e0357033. doi: 10.1371/journal.pone.0357033 (PMC13537555; doi:10.1371/journal.pone.0357033)
Supplement: Peer review history

# Peer review: Decision Letter 0

**DOI:** 10.1371/journal.pone.0357033.r001

**Authors:** Gizachew Tiruneh

28 May 2026

Dear Dr. Muganyizi,

Thank you for submitting your manuscript to PLOS ONE. After careful consideration, we feel that it has merit but does not fully meet PLOS ONE’s publication criteria as it currently stands. Therefore, we invite you to submit a revised version of the manuscript that addresses the points raised during the review process.

The manuscript presents novel empirical evidence relevant to maternal health; however, it requires revision to adequately address the comments raised by both myself and the other reviewer.

Please submit your revised manuscript by Jul 12 2026 11:59PM. If you will need more time than this to complete your revisions, please reply to this message or contact the journal office at plosone@plos.org. When you're ready to submit your revision, log on to https://www.editorialmanager.com/pone/ and select the 'Submissions Needing Revision' folder to locate your manuscript file.

- A letter that responds to each point raised by the academic editor and reviewer(s). You should upload this letter as a separate file labeled 'Response to Reviewers'.
- A marked-up copy of your manuscript that highlights changes made to the original version. You should upload this as a separate file labeled 'Revised Manuscript with Track Changes'.
- An unmarked version of your revised paper without tracked changes. You should upload this as a separate file labeled 'Manuscript'.

If you would like to make changes to your financial disclosure, please include your updated statement in your cover letter. Guidelines for resubmitting your figure files are available below the reviewer comments at the end of this letter.

If applicable, we recommend that you deposit your laboratory protocols in protocols.io to enhance the reproducibility of your results. Protocols.io assigns your protocol its own identifier (DOI) so that it can be cited independently in the future. For instructions see: https://journals.plos.org/plosone/s/submission-guidelines#loc-laboratory-protocols. Additionally, PLOS ONE offers an option for publishing peer-reviewed Lab Protocol articles, which describe protocols hosted on protocols.io. Read more information on sharing protocols at https://plos.org/protocols?utm_medium=editorial-email&utm_source=authorletters&utm_campaign=protocols.

As the corresponding author, your ORCID iD is verified in the submission system and will appear in the published article. PLOS supports the use of ORCID, and we encourage all coauthors to register for an ORCID iD and use it as well. Please encourage your coauthors to verify their ORCID iD within the submission system before final acceptance, as unverified ORCID iDs will not appear in the published article. *Only*  the individual author can complete the verification step; PLOS staff *cannot*  verify ORCID iDs on behalf of authors.

We look forward to receiving your revised manuscript.

Kind regards,

Gizachew Tadele Tiruneh, Ph.D.

Academic Editor

PLOS One

Journal Requirements:

When submitting your revision, we need you to address these additional requirements.

1. Please ensure that your manuscript meets PLOS ONE's style requirements, including those for file naming. The PLOS ONE style templates can be found at

https://journals.plos.org/plosone/s/file?id=wjVg/PLOSOne_formatting_sample_main_body.pdf and

https://journals.plos.org/plosone/s/file?id=ba62/PLOSOne_formatting_sample_title_authors_affiliations.pdf

2. Please include a complete copy of PLOS’ questionnaire on inclusivity in global research in your revised manuscript. Our policy for research in this area aims to improve transparency in the reporting of research performed outside of researchers’ own country or community. The policy applies to researchers who have travelled to a different country to conduct research, research with Indigenous populations or their lands, and research on cultural artefacts. The questionnaire can also be requested at the journal’s discretion for any other submissions, even if these conditions are not met.  Please find more information on the policy and a link to download a blank copy of the questionnaire here: https://journals.plos.org/plosone/s/best-practices-in-research-reporting. Please upload a completed version of your questionnaire as Supporting Information when you resubmit your manuscript.

3. Thank you for stating the following in the Acknowledgments Section of your manuscript:

“We acknowledge the valuable contributions to the WOMAN-2 Trial from the WOMAN-2 collaborators, as well as all colleagues and study participants in Pakistan, Nigeria, Tanzania, and Zambia. The WOMAN-2 trial was funded by The Gates Foundation (INV-007787) and The Wellcome Trust (WT208870/Z/17/Z).”

We note that you have provided funding information that is not currently declared in your Funding Statement. However, funding information should not appear in the Acknowledgments section or other areas of your manuscript. We will only publish funding information present in the Funding Statement section of the online submission form.

Please remove any funding-related text from the manuscript and let us know how you would like to update your Funding Statement. Currently, your Funding Statement reads as follows:

“The WOMAN-2 trial was funded by The Gates Foundation (INV-007787), https://www.gatesfoundation.org and The Wellcome Trust (WT208870/Z/17/Z),  https://wellcome.org to London School of Hygiene and Tropical Medicine (LSHTM). The Funders did not play any role in study design, data collection, decision to publish or preparation of the manuscript.”

Please include your amended statements within your cover letter; we will change the online submission form on your behalf.

4. Thank you for uploading your study's underlying data set. Unfortunately, the repository you have noted in your Data Availability statement does not qualify as an acceptable data repository according to PLOS's standards.

At this time, please upload the minimal data set necessary to replicate your study's findings to a stable, public repository (such as figshare or Dryad) and provide us with the relevant URLs, DOIs, or accession numbers that may be used to access these data. For a list of recommended repositories and additional information on PLOS standards for data deposition, please see https://journals.plos.org/plosone/s/recommended-repositories.

5. Please include captions for your Supporting Information files at the end of your manuscript, and update any in-text citations to match accordingly. Please see our Supporting Information guidelines for more information: http://journals.plos.org/plosone/s/supporting-information.

6. If the reviewer comments include a recommendation to cite specific previously published works, please review and evaluate these publications to determine whether they are relevant and should be cited. There is no requirement to cite these works unless the editor has indicated otherwise.

7. Please review your reference list to ensure that it is complete and correct. If you have cited papers that have been retracted, please include the rationale for doing so in the manuscript text, or remove these references and replace them with relevant current references. Any changes to the reference list should be mentioned in the rebuttal letter that accompanies your revised manuscript. If you need to cite a retracted article, indicate the article’s retracted status in the References list and also include a citation and full reference for the retraction notice.

Additional Editor Comments:

The manuscript presents novel empirical evidence relevant to maternal health; however, it requires revision to adequately address the comments raised by both myself and the other reviewer.

Comments;

Abstract

1) The conclusion appears somewhat overstated. The improvements in hemoglobin, reduced preterm birth, and lower severe anemia at mid-altitudes are supported by the results; however, MAP was lower, which complicates the interpretation of better prenatal health overall. In addition, although blood loss was statistically higher, there was no corresponding difference in the prevalence of PPH. Therefore, it is difficult to conclude that mid-altitude exposure is associated with a clinically meaningful increase in blood loss, and this distinction should be reflected more cautiously in the conclusion.

Methods

2) The manuscript does not clearly follow standard methodological subheadings (e.g., design, data collection, sampling and sample size, and data analysis). In particular, the adequacy of the existing sample powered to answer the stated objectives and how the sampling framework aligns with the altitude comparison is not sufficiently discussed.

3) It is not clearly stated whether women’s altitude exposure was measured at the individual level or assigned as a proxy based on hospital location. If altitude was derived from hospital geographical location, please clarify the rationale and methodological approach used to link hospital altitude to individual maternal exposure during pregnancy and address the potential for exposure misclassification.

4) The analysis of blood loss outcomes does not appear to account for baseline anemia status in the adjustment models, which should be clarified or justified.

Results

5) In Table 1, the chi-square values are not presented

6) The content in Lines 176–181 and 194–198 appears to describe methodological procedures and would be more appropriately placed in the Methods section rather than the Results section.

[Note: HTML markup is below. Please do not edit.]

Reviewers' comments:

Reviewer's Responses to Questions

**Comments to the Author**

1. Is the manuscript technically sound, and do the data support the conclusions?

Reviewer #1: Yes

**********

2. Has the statistical analysis been performed appropriately and rigorously?

Reviewer #1: No

**********

3. Have the authors made all data underlying the findings in their manuscript fully available?

The PLOS Data policy

Reviewer #1: No

**********

4. Is the manuscript presented in an intelligible fashion and written in standard English?

Reviewer #1: Yes

**********

Reviewer #1: Overall Assessment

This study addresses a significant issue in low and middle-income countries: the relationship between maternal outcomes among anemic pregnant women and mid-altitude residence (1000–2499 m). It used data from a large multinational cohort, focused on clinically relevant topic(maternal outcomes), is well organized, has a sound rationale, and has good analysis.

On the other hand, there are some methodological and interpretative concerns that need to be addressed before publication. These include issues related to residual confounding, the assignment of altitude exposure, incomplete accounting for clustering by hospital/country, inconsistencies in statistical reporting, and overinterpretation of small effect sizes as “clinically meaningful.” In general, this manuscript would benefit substantially from clarification of the analytical framework and a more cautious interpretation.

Major Comments

The manuscript states that the women were categorized as having a mid- or low attitude based on the altitude of the hospital where they gave birth. The part of the method section from line number 119-121, including the sentence “The average altitude for each delivery hospital's geographical area was obtained from a free online interactive map program,” confirms this. And the results were repeatedly interpreted as effects of “living” or “residing” at mid-altitude. As someone who grew up and lived in Africa, I knew that women's residential areas and delivery hospital addresses could vary widely because of a lot of reasons: some culture encourage women to give birth in area were they could get better support such as parents house, in-laws house, older sibblings house etc. it is also common for pregnant women with suspected complication outcomes to be referred to hospitals far away from their residential area. Thus, it will be problematic to conclude that the hospital area altitude reflects the women’s residential area. It is better to clarify this and make appropriate modifications.

The other concern is the hierarchical structure and clustering consideration in the analysis. It seems the authors used a standard regression model without multilevel adjustment or clustering. Women are nested within hospitals and countries, yet the analysis appears to use standard regression models without multilevel adjustment or cluster-robust inference. Since altitude is strongly confounded by country/site, and clinical practice and intrapartum interventions differ between hospitals, failure to account for clustering may substantially underestimate standard errors and inflate statistical significance. The manuscript could be benefited from clarify about clustering by hospital/site, using mixed effects or other related models, use hospital/site as a random effect, etc., something that considers different hospitals, sites, and countries

The third major concern is altitude here is likely a proxy for country, residency, hospital type, referral pathways, ethnicity, nutrition, healthcare access, and culture. Although the lack of socioeconomic data is acknowledged in the manuscript, its implications are underestimated. The variable pain control, assisted delivery, oxytocin use, etc., could be affected by the above factors. For example, the 2.5-fold increase in pain medication certainly reflects variation in institutional practice rather than physiologic altitude effects. It would be better to interpret the findings carefully after distinguishing biologically plausible findings from healthcare-system-associated findings. Some explanation of the confounding by facility level clinical practice and adusting for hospital characteristics(if possible) also could benefit the manuscript.

The interpretation of clinical significance of blood loss ( including the phrase “this difference is both statistically and clinically meaningful”), and lower preterm birth risk requires careful revision with evidence-based justifications.

Terminology issue: the incidence rate ratio is used for a cross-sectional binary outcome with a modified Poisson regression analysis. Consider replacing it with the risk ratio(RR). Edite the word “Linear Logistic regression” in table 4.

The phrase: “assumption of normality was not met based on the Z-test” is not clear.

Confusing : “Baseline hemoglobin was 0.63 g/L higher” somewhere else hemoglobin values are reported around 82 g/L. Give it a look.

Minor Comments

The manuscript would benefit from language editing and consistency in phrases like “mid altitude,” “mid-altitude,”“medium altitude.” Asia countries should be Asian countries

**********

what does this mean?). If published, this will include your full peer review and any attached files.

If you choose “no”, your identity will remain anonymous but your review may still be made public.

**Do you want your identity to be public for this peer review?**  For information about this choice, including consent withdrawal, please see our Privacy Policy

Reviewer #1: **Yes:**  Meselech Ambaw Dessie

**********

[NOTE: If reviewer comments were submitted as an attachment file, they will be attached to this email and accessible via the submission site. Please log into your account, locate the manuscript record, and check for the action link "View Attachments". If this link does not appear, there are no attachment files.]

To ensure your figures meet our technical requirements, please review our figure guidelines: https://journals.plos.org/plosone/s/figures

You may also use PLOS’s free figure tool, NAAS, to help you prepare publication quality figures: https://journals.plos.org/plosone/s/figures#loc-tools-for-figure-preparation.

NAAS will assess whether your figures meet our technical requirements by comparing each figure against our figure specifications.

# Author comment: Author response to Decision Letter 1

**DOI:** 10.1371/journal.pone.0357033.r002

26 Jun 2026

1)Please ensure that your manuscript meets PLOS ONE's style requirements, including those for file naming. R.[We have complied].

2).Please include a complete copy of PLOS’ questionnaire on inclusivity in global research in your revised manuscript. Please upload a completed version of your questionnaire as Supporting Information when you resubmit your manuscript. R:[A completed version has been uploaded as supporting evidence]

3).Please remove any funding-related text from the manuscript and let us know how you would like to update your Funding Statement. Currently, your Funding Statement reads as follows:

“The WOMAN-2 trial was funded by The Gates Foundation (INV-007787), https://www.gatesfoundation.org and The Wellcome Trust (WT208870/Z/17/Z), https://wellcome.org to London School of Hygiene and Tropical Medicine (LSHTM). The Funders did not play any role in study design, data collection, decision to publish or preparation of the manuscript.” Please include your amended statements within your cover letter; we will change the online submission form on your behalf.R:[Funding information has been deleted from the acknowledgement;The statement has been included in the cover letter, which is the same as in the Funding Statement section of the current online submission]

3)Thank you for uploading your study's underlying data set. Unfortunately, the repository you have noted in your Data Availability statement does not qualify as an acceptable data repository according to PLOS's standards.

At this time, please upload the minimal data set necessary to replicate your study's findings to a stable, public repository (such as figshare or Dryad) and provide us with the relevant URLs, DOIs, or accession numbers that may be used to access these data. For a list of recommended repositories and additional information on PLOS standards for data deposition, please see https://journals.plos.org/plosone/s/recommended-repositories. R:[Minimal data has been uploaded to the FIGSHARE Repository: https://doi.org/10.6084/m9.figshare.32794914].

5).Please include captions for your Supporting Information files at the end of your manuscript, and update any in-text citations to match accordingly. Please see our Supporting Information guidelines for more information: http://journals.plos.org/plosone/s/supporting-information.R[Captions included: •S1Table.Hospitals with corresponding altitude values and the number of participants;•S2Table. Maternal and labour characteristics in the low-and mid-land. Presented as N(%) unless otherwise indicated;•S3Checklist. Inclusivity in Global Health Research]

6).If the reviewer comments include a recommendation to cite specific previously published works, please review and evaluate these publications to determine whether they are relevant and should be cited. There is no requirement to cite these works unless the editor has indicated otherwise. R:[No new citation recommendation was received. Supporting information is available at: https://doi.org/10.6084/m9.figshare.32794914]

7).Please review your reference list to ensure that it is complete and correct. If you have cited papers that have been retracted, please include the rationale for doing so in the manuscript text, or remove these references and replace them with relevant current references. R:[References Checked for Retraction. No retracted reference was found.]

ADDITIONAL EDITORIAL COMMENTS:

8).The conclusion appears somewhat overstated. The improvements in hemoglobin, reduced preterm birth, and lower severe anemia at mid-altitudes are supported by the results; however, MAP was lower, which complicates the interpretation of better prenatal health overall. In addition, although blood loss was statistically higher, there was no corresponding difference in the prevalence of PPH. Therefore, it is difficult to conclude that mid-altitude exposure is associated with a clinically meaningful increase in blood loss, and this distinction should be reflected more cautiously in the conclusion. R:[We reviewed the analysis by accounting for clustering at the hospital level and including country as a fixed effect, as appropriate for modeling dichotomous and continuous outcomes. From the reanalysis:

•The significance of lower MAP persisted, which means a lower risk of cardiovascular disease, thus supporting overall better prenatal health and a lower risk of cardiovascular disease, such as pre-eclampsia among women in mid-altitude areas.•After the reanalysis, mid-altitude was not independently associated with increased blood loss, hence the conclusion has been revised accordingly.

9).Methods:The manuscript does not clearly follow standard methodological subheadings (e.g., design, data collection, sampling and sample size, and data analysis). In particular, the adequacy of the existing sample powered to answer the stated objectives and how the sampling framework aligns with the altitude comparison is not sufficiently discussed.In particular, the adequacy of the existing sample powered to answer the stated objectives and how the sampling framework aligns with the altitude comparison is not sufficiently discussed.R:[The Materials and methods section and the manuscript as a whole has been reformatted to comply with PLOS requirements, including sample size calculations: L91-217].

10).It is not clearly stated whether women’s altitude exposure was measured at the individual level or assigned as a proxy based on hospital location. If altitude was derived from hospital geographical location, please clarify the rationale and methodological approach used to link hospital altitude to individual maternal exposure during pregnancy, and address the potential for exposure misclassification. R: [•Women’s altitude exposure was based on the mean altitude at the delivery hospital's geographic location, as explained in the Materials and Methods (L125-L131).•Further, it has been acknowledged that delivery altitude could differ from the residential altitude, since most such deliveries come from the hospital catchment area; we reasonably took it as a proxy for individual maternal residential exposure (L365-L371).•The entire document has been revised to read as delivery altitude rather than residential altitude.]

11).The analysis of blood loss outcomes does not appear to account for baseline anemia status in the adjustment models, which should be clarified or justified.R: [True, we did not control for anemia because Anemia was a criterion for enrolment of all the women in the study. All women in this study were anemic.]

12).Results: In Table 1, the chi-square values are not presented. R:[•We have revised Table 1 to include the chi-square/t-test p-values where appropriate as advised (Table 1)].

13).The content in Lines 176–181 and 194–198 appears to describe methodological procedures and would be more appropriately placed in the Methods section rather than the Results section.R:[•This has been revised such that all the misplaced methodological information has been relocated to the Materials and Methods section (L185-L310)].

REVIEWER #1

1).The manuscript states that the women were categorized as having a mid- or low attitude based on the altitude of the hospital where they gave birth. The part of the method section from line number 119-121, including the sentence “The average altitude for each delivery hospital's geographical area was obtained from a free online interactive map program,” confirms this. And the results were repeatedly interpreted as effects of “living” or “residing” at mid-altitude. As someone who grew up and lived in Africa, I knew that women's residential areas and delivery hospital addresses could vary widely because of a lot of reasons: some culture encourage women to give birth in area were they could get better support such as parents house, in-laws house, older sibblings house etc. it is also common for pregnant women with suspected complication outcomes to be referred to hospitals far away from their residential area. Thus, it will be problematic to conclude that the hospital area altitude reflects the women’s residential area. It is better to clarify this and make appropriate modifications. R: [•We have revised the entire document to ensure the altitudes are referred to as places of delivery/birth rather than the places of residence;•In the discussion section, the altitudes for hospital location are discussed as a proxy to residencial altitude since most participants who gave birth in a hospital are expected to reside within the hospital catchment area. However, this assumption is acknowledged in the study limitations paragraph and suggested for future studies to use the individual participant’s residential location altitude for better outcomes (L365-L372)].

2) The other concern is the hierarchical structure and clustering consideration in the analysis. It seems the authors used a standard regression model without multilevel adjustment or clustering. Women are nested within hospitals and countries, yet the analysis appears to use standard regression models without multilevel adjustment or cluster-robust inference. Since altitude is strongly confounded by country/site, and clinical practice and intrapartum interventions differ between hospitals, failure to account for clustering may substantially underestimate standard errors and inflate statistical significance. The manuscript could be benefited from clarify about clustering by hospital/site, using mixed effects or other related models, use hospital/site as a random effect, etc., something that considers different hospitals, sites, and countries. R:[•The analysis has been revised, taking into consideration the hierarchical structure and clustering (cluster 1: Individual participant; Cluster 2: Country; Cluster 3: Hospital site);•Based on the hierarchy, we applied classical logistic regression and modified Poisson regression with cluster-robust standard errors, accounting for clustering at the hospital level and including country as a fixed effect to model dichotomous outcomes (L185-L199);•The analysis is justified since we have a good number of hospital sites (#42), and our interest was to apply the results to the population rather than studying variations among hospitals;•Likewise, for continuous outcome variables, we performed linear regression with cluster-robust variance at the hospital level and country fixed effects;•The analysis is explained in the Materials and Methods section (L185-L199), and the revised results are in Tables 3 & 4.]

3)The third major concern is altitude here is likely a proxy for country, residency, hospital type, referral pathways, ethnicity, nutrition, healthcare access, and culture. Although the lack of socioeconomic data is acknowledged in the manuscript, its implications are underestimated. The variable pain control, assisted delivery, oxytocin use, etc., could be affected by the above factors. For example, the 2.5-fold increase in pain medication certainly reflects variation in institutional practice rather than physiologic altitude effects. It would be better to interpret the findings carefully after distinguishing biologically plausible findings from healthcare-system-associated findings. Some explanation of the confounding by facility level clinical practice and adusting for hospital characteristics(if possible) also could benefit the manuscript; The interpretation of clinical significance of blood loss ( including the phrase “this difference is both statistically and clinically meaningful”), and lower preterm birth risk requires careful revision with evidence-based justifications.

Terminology issue: the incidence rate ratio is used for a cross-sectional binary outcome with a modified Poisson regression analysis. Consider replacing it with the risk ratio(RR). Edite the word “Linear Logistic regression” in table 4;The phrase: “assumption of normality was not met based on the Z-test” is not clear.

R: [•The analysis has been revised as explained above to take care of the confounding effect by country and hospital;• In the reanalysis, some residual confounders have been controlled. However, socioeconomic variables were not available; hence, the acknowledgement is made in the limitation section (L356-L399);•After the reanalysis above, blood loss was not independently associated with altitude level; hence, its discussion has been revised.

•The low preterm birth rate in mid-altitudes in the current study could be linked to altitude. The association Lof altitude with preterm birth has resulted in inconsistent findings in previous studies as explained in the discussion section (L297-L305);•These terminologies have been revised. The incidence rate ratio have been correctly replaced by Risk ratios (RR). “Linear Logistic regression” has been corrected to read “linear regression” (L185-199).

•The phrase “assumption of normality was not met based on the Z-test” has been corrected to read as “assumption of normality was not met.” (L178-L184).

4). Confusing : “Baseline hemoglobin was 0.63 g/L higher” somewhere else hemoglobin values are reported around 82 g/L. Give it a look.

Minor Comments

The manuscript would benefit from language editing and consistency in phrases like “mid altitude,” “mid-altitude,”“medium altitude.” Asia countries should be Asian countries. R:[•The language has been corrected throughout the document. Mid altitude is termed mid-altitude and low altitude as low-altitude throughout the document].

Attachment Submitted filename: Response to Reviewers.docx

# Peer review: Decision Letter 1

**DOI:** 10.1371/journal.pone.0357033.r003

**Authors:** Gizachew Tiruneh

24 Jul 2026

Dear Dr. Muganyizi,

Please submit your revised manuscript by Sep 07 2026 11:59PM. If you will need more time than this to complete your revisions, please reply to this message or contact the journal office at plosone@plos.org. When you're ready to submit your revision, log on to https://www.editorialmanager.com/pone/ and select the 'Submissions Needing Revision' folder to locate your manuscript file.

- A letter that responds to each point raised by the academic editor and reviewer(s). You should upload this letter as a separate file labeled 'Response to Reviewers'.
- A marked-up copy of your manuscript that highlights changes made to the original version. You should upload this as a separate file labeled 'Revised Manuscript with Track Changes'.
- An unmarked version of your revised paper without tracked changes. You should upload this as a separate file labeled 'Manuscript'.

If you would like to make changes to your financial disclosure, please include your updated statement in your cover letter. Guidelines for resubmitting your figure files are available below the reviewer comments at the end of this letter.

If applicable, we recommend that you deposit your laboratory protocols in protocols.io to enhance the reproducibility of your results. Protocols.io assigns your protocol its own identifier (DOI) so that it can be cited independently in the future. For instructions see: https://journals.plos.org/plosone/s/submission-guidelines#loc-laboratory-protocols. Additionally, PLOS ONE offers an option for publishing peer-reviewed Lab Protocol articles, which describe protocols hosted on protocols.io. Read more information on sharing protocols at https://plos.org/protocols?utm_medium=editorial-email&utm_source=authorletters&utm_campaign=protocols.

As the corresponding author, your ORCID iD is verified in the submission system and will appear in the published article. PLOS supports the use of ORCID, and we encourage all coauthors to register for an ORCID iD and use it as well. Please encourage your coauthors to verify their ORCID iD within the submission system before final acceptance, as unverified ORCID iDs will not appear in the published article. *Only*  the individual author can complete the verification step; PLOS staff *cannot*  verify ORCID iDs on behalf of authors.

We look forward to receiving your revised manuscript.

Kind regards,

Gizachew Tadele Tiruneh, Ph.D.

Academic Editor

PLOS One

Journal Requirements:

If the reviewer comments include a recommendation to cite specific previously published works, please review and evaluate these publications to determine whether they are relevant and should be cited. There is no requirement to cite these works unless the editor has indicated otherwise.

Please review your reference list to ensure that it is complete and correct. If you have cited papers that have been retracted, please include the rationale for doing so in the manuscript text, or remove these references and replace them with relevant current references. Any changes to the reference list should be mentioned in the rebuttal letter that accompanies your revised manuscript. If you need to cite a retracted article, indicate the article’s retracted status in the References list and also include a citation and full reference for the retraction notice.

[Note: HTML markup is below. Please do not edit.]

Reviewers' comments:

Reviewer's Responses to Questions

**Comments to the Author**

Reviewer #1: All comments have been addressed

**********

2. Is the manuscript technically sound, and do the data support the conclusions?

Reviewer #1: Yes

**********

3. Has the statistical analysis been performed appropriately and rigorously?

Reviewer #1: Yes

**********

4. Have the authors made all data underlying the findings in their manuscript fully available?

The PLOS Data policy

Reviewer #1: Yes

**********

5. Is the manuscript presented in an intelligible fashion and written in standard English?

Reviewer #1: Yes

**********

Reviewer #1: The authors have adequately addressed the major methodological concerns I highlighted in the previous review: clarified the use of hospital altitude as a proxy exposure, incorporated clustering in the regression analyses, revised the statistical methods, and substantially moderated the interpretation of their findings.

I have only a few remaining minor comments regarding

1. Covariate selection description

It says “Covariates included in the multivariable regression models were selected based on statistical significance in the bivariate analyses (p ≤ 0.05) and their established clinical relevance...” Could you revise this wording? It's not the best way to describe it.

2. Residual confounding

Altitude remains strongly correlated with country. Some countries are either mainly mid-altitude or low-altitude. Could you discuss this limitation a little more explicitly?

3. Clarification of the sample size

From my understanding, no additional sample size calculation was performed because this was a secondary analysis of an existing dataset. Could you clarify that, please?

4. Typo errors including missing spaces, letters, and some inconsistent capitalizations

These issues are minor and can be addressed without additional analyses.

**********

what does this mean?). If published, this will include your full peer review and any attached files.

If you choose “no”, your identity will remain anonymous but your review may still be made public.

**Do you want your identity to be public for this peer review?**  For information about this choice, including consent withdrawal, please see our Privacy Policy

Reviewer #1: No

**********

[NOTE: If reviewer comments were submitted as an attachment file, they will be attached to this email and accessible via the submission site. Please log into your account, locate the manuscript record, and check for the action link "View Attachments". If this link does not appear, there are no attachment files.]

To ensure your figures meet our technical requirements, please review our figure guidelines: https://journals.plos.org/plosone/s/figures

You may also use PLOS’s free figure tool, NAAS, to help you prepare publication quality figures: https://journals.plos.org/plosone/s/figures#loc-tools-for-figure-preparation.

NAAS will assess whether your figures meet our technical requirements by comparing each figure against our figure specifications.

# Author comment: Author response to Decision Letter 2

**DOI:** 10.1371/journal.pone.0357033.r004

31 Jul 2026

POINT BY POINT RESPONSES TO REVIEWERS

A. Editorial Points

1. Please upload a Response to Reviewers letter which should include a point by point response to each of the points made by the Editor and / or Reviewers. (This should be uploaded as a 'Response to Reviewers' file type.) Please follow this link for more information: http://blogs.PLOS.org/everyone/2011/05/10/how-to-submit-your-revised-manuscript/

Response: The Response to Reviewers letter has been uploaded, which includes our responses to points by the Editor and Reviewer #1.

2. It is important that you include a cover letter with your manuscript. Please ensure that this letter is addressed specifically to PLoS ONE. A cover letter has been included that addresses the following:

* why this manuscript is suitable for publication in PLoS ONE.

* how does your paper provide a worthwhile addition to the scientific literature?

* how does your paper relate to previously published work?

* which types of scientists do you believe will be most interested in your study?

Response: A cover letter has been uploaded with details of the outlined points.

B. Reviewer 1

1. Covariate selection description

It says “Covariates included in the multivariable regression models were selected based on statistical significance in the bivariate analyses (p ≤ 0.05) and their established clinical relevance...” Could you revise this wording? It's not the best way to describe it.

Response: Thank you for the comment. The statement has been refined to read as, “Covariates incorporated into the multivariable regression models were selected on the basis of statistical significance observed in bivariate analyses (p ≤ 0.05), as well as their established clinical relevance, substantiated by prior literature and expert consensus.”[L196-199]

2. Residual confounding

Altitude remains strongly correlated with country. Some countries are either mainly mid-altitude or low-altitude. Could you discuss this limitation a little more explicitly?

Response: We agree with the observation. We have added a statement that reads as, “Moreover, recognizing that the study countries differed in landscape characteristics, we adjusted for country in the multivariable analyses to mitigate potential confounding” to the limitations in the Discussion section [L374-377]

3. Clarification of the sample size

From my understanding, no additional sample size calculation was performed because this was a secondary analysis of an existing dataset. Could you clarify that, please?

Response: We have added a short sentence to the Sample Size Calculation subsection which states: “The current analysis was based on the WOMAN-2 dataset” [L106].

4. Typo errors including missing spaces, letters, and some inconsistent capitalizations

Response: The document was cross-checked for typos and corrected.

5. These issues are minor and can be addressed without additional analyses.

Response: Noted.

Sincerely,

Projestine Muganyizi

Correspondent Author

Attachment Submitted filename: Response to Reviewers file.docx

# Editor report: Decision Letter 2

**DOI:** 10.1371/journal.pone.0357033.r005

**Authors:** Gizachew Tiruneh

12 Aug 2026

Maternal outcomes at mid-altitudes in anemic pregnant women: Evidence from the  WOMAN-2 trial.

PONE-D-25-47970R2

Dear Dr. Muganyizi,

We’re pleased to inform you that your manuscript has been judged scientifically suitable for publication and will be formally accepted for publication once it meets all outstanding technical requirements.

Within one week, you’ll receive an e-mail detailing the required amendments. When these have been addressed, you’ll receive a formal acceptance letter and your manuscript will be scheduled for publication.

An invoice will be generated when your article is formally accepted. Please note, if your institution has a publishing partnership with PLOS and your article meets the relevant criteria, all or part of your publication costs will be covered. Please make sure your user information is up-to-date by logging into Editorial Manager at Editorial Manager® and clicking the ‘Update My Information' link at the top of the page. For questions related to billing, please contact billing support.

If your institution or institutions have a press office, please notify them about your upcoming paper to help maximize its impact. If they’ll be preparing press materials, please inform our press team as soon as possible -- no later than 48 hours after receiving the formal acceptance. Your manuscript will remain under strict press embargo until 2 pm Eastern Time on the date of publication. For more information, please contact onepress@plos.org.

Kind regards,

Gizachew Tadele Tiruneh, Ph.D.

Academic Editor

PLOS One

Additional Editor Comments (optional):

Reviewers' comments:

# Editor report: Acceptance letter

**DOI:** 10.1371/journal.pone.0357033.r006

**Authors:** Gizachew Tiruneh

PONE-D-25-47970R2

PLOS One

Dear Dr. Muganyizi,

I'm pleased to inform you that your manuscript has been deemed suitable for publication in PLOS One. Congratulations! Your manuscript is now being handed over to our production team.

At this stage, our production department will prepare your paper for publication. This includes ensuring the following:

* All references, tables, and figures are properly cited

* All relevant supporting information is included in the manuscript submission,

* There are no issues that prevent the paper from being properly typeset

You will receive further instructions from the production team, including instructions on how to review your proof when it is ready. Please keep in mind that we are working through a large volume of accepted articles, so please give us a few days to review your paper and let you know the next and final steps.

Lastly, if your institution or institutions have a press office, please let them know about your upcoming paper now to help maximize its impact. If they'll be preparing press materials, please inform our press team within the next 48 hours. Your manuscript will remain under strict press embargo until 2 pm Eastern Time on the date of publication. For more information, please contact onepress@plos.org.

You will receive an invoice from PLOS for your publication fee after your manuscript has reached the completed accept phase. If you receive an email requesting payment before acceptance or for any other service, this may be a phishing scheme. Learn how to identify phishing emails and protect your accounts at https://explore.plos.org/phishing.

If we can help with anything else, please email us at customercare@plos.org.

Thank you for submitting your work to PLOS One and supporting open access.

Kind regards,

PLOS One Editorial Office Staff

on behalf of

Dr. Gizachew Tadele Tiruneh

Academic Editor

PLOS One
